# Osteoblasts impair cholesterol synthesis in chondrocytes via Notch1 signalling

**DOI:** 10.1111/cpr.13156

**Published:** 2021-11-02

**Authors:** Yueyi Yang, Demao Zhang, Daimo Guo, Jiachi Li, Siqun Xu, Jieya Wei, Jing Xie, Xuedong Zhou

**Affiliations:** ^1^ State Key Laboratory of Oral Diseases West China Hospital of Stomatology Sichuan University Chengdu China; ^2^ Department of Cariology and Endodontics West China Hospital of Stomatology Sichuan University Chengdu China

**Keywords:** cholesterol, chondrocytes, Notch, osteoblasts, seladin‐1

## Abstract

**Objectives:**

Previous reports have proposed the importance of signalling and material exchange between cartilage and subchondral bone. However, the specific experimental evidence is still insufficient to support the effect of this interdependent relationship on mutual cell behaviours. In this study, we aimed to investigate cellular lipid metabolism in chondrocytes induced by osteoblasts.

**Methods:**

Osteoblast‐induced chondrocytes were established in a Transwell chamber. A cholesterol detection kit was used to detect cholesterol contents. RNA sequencing and qPCR were performed to assess changes in mRNA expression. Western blot analysis was performed to detect protein expression. Immunofluorescence staining was conducted to show the cellular distribution of proteins.

**Results:**

Cholesterol levels were significantly decreased in chondrocytes induced by osteoblasts. Osteoblasts reduced cholesterol synthesis in chondrocytes by reducing the expression of a series of synthetases, including Fdft1, Sqle, Lss, Cyp51, Msmo1, Nsdhl, Sc5d, Dhcr24 and Dhcr7. This modulatory process involves Notch1 signalling. The expression of ncstn and hey1, an activator and a specific downstream target of Notch signalling, respectively, were decreased in chondrocytes induced by osteoblasts.

**Conclusions:**

For the first time, we elucidated that communication with osteoblasts reduces cholesterol synthesis in chondrocytes through Notch1 signalling. This result may provide a better understanding of the effect of subchondral bone signalling on chondrocytes.

## Graphical abstract

Osteoblasts inhibited the expression of seladin‐1 and Notch signalling in chondrocytes, thereby reducing the cholesterol synthesis in chondrocytes.

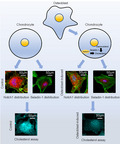

## 1 INTRODUCTION

Articular cartilage, calcified cartilage, subchondral bone, cortical bone and cancellous bone form a biological complex in the bone and joint referred to as the osteochondral unit. Chondrocytes are unique cell types in articular cartilage tissue and are essential for cartilage formation and function. Osteoblasts are the main components of subchondral bone. Osteoblasts regulate chondrocyte behaviour.^1^, ^2^, ^3^

The lipid components of biological membranes are important for normal cellular functions.^4^ Changes in lipid tissue may exert profound effects on cellular functions, such as signal transduction and membrane trafficking.^5^, ^6^, ^7^ Cholesterol is one of the most important regulators of lipid tissue, and mammals have developed complex mechanisms to maintain intracellular cholesterol levels within a narrow range.^8^ When these homeostatic mechanisms are overwhelmed, various diseases, such as atherosclerosis, occur. The role of lipids in various diseases has been studied for decades.^9^ The association between lipids and chondrocytes has become an increasing focus of research interest in recent years. The lipidic microenvironment in human chondrocytes induces oxidative stress and elicits a proinflammatory response, which might be reflected in joint diseases.^10^ Shabana et al. reported that the activation of HH signalling in chondrocytes induces cholesterol accumulation, which increases the severity of osteoarthritis (OA) in transgenic mice.^11^

The Notch signalling pathway is crucial for the determination of cell‐to‐cell communication and cell fate during development and is necessary for tissue homeostasis. In skeletal muscle and brain, activation of the Notch signalling pathway enforces the resting state of local adult stem cells, thereby limiting their tissue repair potential and subsequently affecting metabolism.^12^, ^13^ In the immune system, activation of Notch signalling promotes the polarization of M1 macrophages, induces a systemic low‐grade inflammatory state, and aggravates insulin resistance in peripheral tissues.^14^, ^15^ In addition, Notch stabilizes and activates mTorc1,^16^ which plays a central role in lipid metabolism,^17^ resulting in increased de novo lipogenesis and fatty liver. Notch signalling pathways are complex and involve four Notch receptors (Notch1–4). Garces et al. first showed that Notch1 is inseparable for adipogenesis.^17^ However, Notch signalling has rarely been studied in chondrocytes.

In the current study, we aimed to study the effect of osteoblast‐induced culture on chondrocyte lipid metabolism using osteoblast‐induced chondrocytes and chondrocyte monoculture models. We found that osteoblasts reduced the cholesterol content and the expression of the cholesterol synthase seladin‐1 by performing qPCR, WB and IF. Additionally, osteoblasts inhibited cholesterol synthesis in chondrocytes through Notch1 signalling. This study will help us further understand how osteoblast‐derived signals regulate chondrocyte behaviour.

## 2 MATERIALS AND METHODS

### 2.1 Cell culture

This study strictly abided by ethical principles when using animal cells. All experimental protocols described in our study were reviewed and approved by the Institutional Review Board (West China Hospital of Stomatology, No. WCHSIRB‐D‐2021‐014) at the State Key Laboratory of Oral Diseases, West China Hospital of Stomatology, Sichuan University.

Articular chondrocytes and primary calvarial osteoblasts were isolated from C57BL/6J mice 2–3 days after birth. Briefly, the articular cartilage and skull bone were dissected from the mice, cut into pieces, and enzymatically digested. First, the tissue was trypsinized with a 0.25% protease solution dissolved in Dulbecco's modified Eagle's medium (HyClone) for 30 min. Then, chondrocytes were digested with 0.003% type II collagenase (Sigma–Aldrich) for 12 h, and osteoblasts were digested with 0.006% type I collagenase (Sigma–Aldrich) for 12 h. After centrifugation at 1000 rpm for 8 min, the supernatant was removed, and complete supplemental medium composed of either DMEM, 10% FBS and 1% penicillin‐streptomycin or α‐MEM, 10% FBS and 1% penicillin‐streptomycin was mixed with the two types of cells and tissues. Then, the primary chondrocytes and osteoblasts were seeded into a 25 cm^2^ cell culture flask and cultivated at 37 °C with 5% CO_2_ in a standard humidified atmosphere. Osteoblasts and chondrocytes were used at the second passage in the current study.

Chondrocytes were seeded into a six‐well plate, and osteoblasts were seeded on a Transwell chamber with a 0.4 μm pore size to construct a coculture model of osteoblast‐induced chondrocytes and chondrocyte monocultures. After the cells adhered, chondrocytes and osteoblasts were equilibrated with DMEM/α‐MEM containing 10% FBS for 12 h, substituted with DMEM/α‐MEM containing 2% FBS, and starved for 12 h. When finally changing the medium to DMEM/α‐MEM containing 1% FBS, the Transwell chamber was placed in a six‐well plate. After culture for 3 days, cell lysates (1000 ml) were collected. The cell confluence at the beginning of the coculture was approximately 60%, which was still in the logarithmic growth phase.

### 2.2 ELISA

We collected chondrocyte culture medium from osteoblast‐induced chondrocytes and chondrocyte monoculture models after 72 h. ELISA kits (CSB‐EL001918MO; CSB‐EL001712MO, CUSABIO) were used to detect apolipoprotein B and angiopoietin‐like 4 levels according to the instruction manual.

### 2.3 RNA sequencing

Chondrocytes were cultured in the osteoblast induction system and the monoculture system for 12 h and then incubated with 2% FBS and 1% penicillin‐streptomycin. The cells were starved in DMEM for 12 h. Then, the medium was changed to DMEM containing 1% FBS and 1% penicillin‐streptomycin and cells were cultivated for 72 h. TRIzol was used to collect cell lysates (cell confluence rate of up to 95%). Three independent replicate experiments were performed using chondrocytes extracted from the knee joints of different postnatal C57 mice. The cell samples were sent to Shanghai Lifegenes Biotechnology Co., Ltd. for the transcriptome analysis. Before transcriptome sequencing, we used the RNA Nano 6000 Assay Kit and the Bioanalyzer 2100 system to evaluate the RNA integrity. According to the manufacturer's instructions, each sample used 1.5 μg of RNA as the input material for RNA detection. A HiSeq 4000 PE Cluster Kit (Illumina) was used to cluster the index‐coded samples. The original data were first processed with internal scripts to obtain clean data and then compared with the reference genome using HISAT2 v2.1.0. During data analysis, HTSeq v0.6.1 was employed to calculate the number of reads mapped to each gene. The method used to calculate gene FPKMs was to add the FPKMs of each genomic transcript. The GO enrichment analysis of differentially expressed genes was performed using the DAVID database. GO terms with a *p* value less than 0.05 were considered observably enriched in differentially expressed genes. KEGG is a database and resource for the public to understand the advanced functions in biological systems. KOBAS v3.0 software was used to detect the statistically significant enrichment of differentially expressed genes in various KEGG pathways. Differentially expressed genes were significantly enriched for KEGG pathways with a *p* value less than 0.05 (adjusted *p* values were used to screen different candidate genes). In the differential expression analysis, *p* < 0.05 and |FoldChange| ≥ 1.2 were the thresholds for significantly different expression.

### 2.4 RNA extraction and quantitative real‐time PCR

The RNeasyPlus Mini Kit (Qiagen) was used to extract total RNA from chondrocytes. Then, a cDNA synthesis kit (K1621‐RevertAid, Mbi) was used to purify the extracted RNA sample and reverse transcribe it into cDNAs according to the manufacturer's instructions. The SYBR Premix Ex Taq II PCR Kit (TAKARA) was used to perform qPCR on a Bio‐Rad CFX Manager instrument. The cycle threshold (Ct) value of the sample was normalized to the value of the GAPDH housekeeping gene, and the relative expression was calculated using the 2^−ΔΔCt^ method. The primer sequences are shown in Table 1.

**TABLE 1:** Primer pairs designed in the study

mRNA	Primer pairs
GAPDH (87 bp)	Forward: GGGTCCCAGCTTAGGTTCATC Reverse: AATCCGTTCACACCGACCTT
β‐ACTIN (200 bp)	Forward: ACTGAGCTGCGTTTTACACC Reverse: GCCTTCACCGTTCCAGTTTTT
Dhcr24 (200 bp)	Forward: CCGTCCATCAGTGGTCCTTC Reverse: GGTGATTCTGCCTTCCCACA
Fdft1 (180 bp)	Forward: GTGAGGCCACGTGTGATGG Reverse: AGCATCGCGCAGAACAACAC
Sqle (172 bp)	Forward: CGCAGCGGTTACTCTGGTTA Reverse: ATTCCTCCTCAAGCAAGCCC
Lss (151 bp)	Forward: AGGCTGTCAGGTAAAGGGCT Reverse: CTGCCGACCCAACTCATTCT
Cyp51 (97 bp)	Forward: CACACATTGCCACAGGGAGA Reverse: GAAGTGGCCCAACTACACGA
Msmol1 (79 bp)	Forward: AAGGTTTCGGGAACTGGAGG Reverse: AAGGCAACGTCAACTTCAGC
Nsdhl (161 bp)	Forward: CGTCCTCATGGCATTTTCGG Reverse: TCAGCGGCTAAGATGTGTCC
Dhcr7 (127 bp)	Forward: GCACAGTCCCTGGCTAACTT Reverse: GTTGTCCGAGCCAAGTCTCA
Sc5d (190 bp)	Forward: GCGCTCTGGCTCGTACTCT Reverse: GAAGTAGTAATCGGCGGCAC
Tm7sf2 (191 bp)	Forward: GTCGCGGCTTTACTGATCCT Reverse: CGTGCAGGCAGCAAATAGAG
Ebp (104 bp)	Forward: ATGGGTCCGGGAACTGATTG Reverse: TCGCACAAGATGAGGCTGAA
Cyp2r10 (185 bp)	Forward: TGGAGGCATATCAACTGTCGT Reverse: TGTTAACAGCCAACCTTCTGTG
Cel (131 bp)	Forward: ACTACCTGGCCTTCATCCCT Reverse: GGTAGCAAATAGGTGGCCGT
Soat1 (138 bp)	Forward: GCCTTGTGCGACTTATGTGTT Reverse: TCTAACCCGAGGCAAGCAA
Hsd17b7 (141 bp)	Forward: ACTGTGACACCGTACAACG Reverse: TATGTCCATCTTTTGGCCCGT
SMT1 (102 bp)	Forward: CACTCAGTAGCTTCCGCCTT Reverse: CCCTTTGGCTTCCTTGAGGT
SMT2 (98 bp)	Forward: AAAGGCAAACGAGCCGTAGA Reverse: TTTGGACGGCGGAAGAAAGA

### 2.5 Western blotting

Cells were lysed with RIPA buffer (Pierce) on ice to prepare total cellular protein. The sample was denatured with an equal volume of Bio‐Rad Laemmli sample buffer (Bio‐Rad Laboratories) at 100°C for 5 min to obtain the final protein sample. The protein samples were separated on 10% SDS–PAGE gels and then transferred to polyvinylidene fluoride (PVDF) membranes. The membrane was blocked with a Tris buffer solution containing 0.05% Tween 20 and 5% skim milk powder for 1 h and then incubated with the appropriate primary antibody (anti‐mouse β‐actin, 1:2000, sc‐47778, Abcam; anti‐rabbit Seladin‐1, 1:3000, ab40490, Abcam; anti‐rabbit Notch1, 1:1000, 380355; hey1, 1:1000, 516110; ncstn, 1:1000, 500128; Zen‐Bio) overnight at 4°C. Membranes were washed with TBST, and the homologous secondary antibody (anti‐mouse, m‐IgGКBP‐HRP, 1:4000, sc‐516102; anti‐rabbit, IgG‐HRP, 1:2000, sc‐2357, Abcam) was incubated with the membrane for 2 h. The Immobilon^®^ Western (P90719, Millipore) kit was used to visualize immune complexes, and the protein expression levels were analysed with ImageJ software (NIH). In addition, β‐actin was used as an internal control.

### 2.6 Immunofluorescence staining

Chondrocytes were cultured as described above for 72 h in a cell culture dish designed for laser confocal microscopy. Then, the cells were washed three times with 1 × PBS, fixed with 4% paraformaldehyde (PFA) for 10 min and rinsed with PBS three times. After permeabilization with 0.25% Triton X‐100 for 10 min, the cells were blocked with 5% BSA for 1 h. The samples were then incubated with rabbit monoclonal antibodies (Seladin‐1, 1:200; Abcam; Notch1, 1:200; ncstn, 1:200, hey1, 1:200; Zen‐Bio) overnight at 4°C. Next, the samples were incubated with a secondary antibody conjugated to Alexa Fluor 647 (ab150075; Abcam) for 2 h. DAPI (D9542; Sigma–Aldrich) and phalloidin (6 μmol/L; Invitrogen) were used to stain the nucleus and cytoskeleton, respectively. Then, the cells were observed under a confocal laser scanning microscope.

The Cholesterol Assay Kit (Cell‐Based; Abcam) was used to observe changes in the cholesterol content.

### 2.7 Statistical analysis

The results of each analysis are presented as the means ± SD. Experiments were performed in triplicate (*n* = 3). Statistical analyses were performed using one‐way analysis of variance to determine differences between groups. Fisher's protected least effective difference test was performed as a post hoc analysis. In each analysis, the critical significance level was set to *p* < 0.05.

## 3 RESULTS

### 3.1 Osteoblasts reduce the intracellular cholesterol content in chondrocytes

**FIGURE 1:**
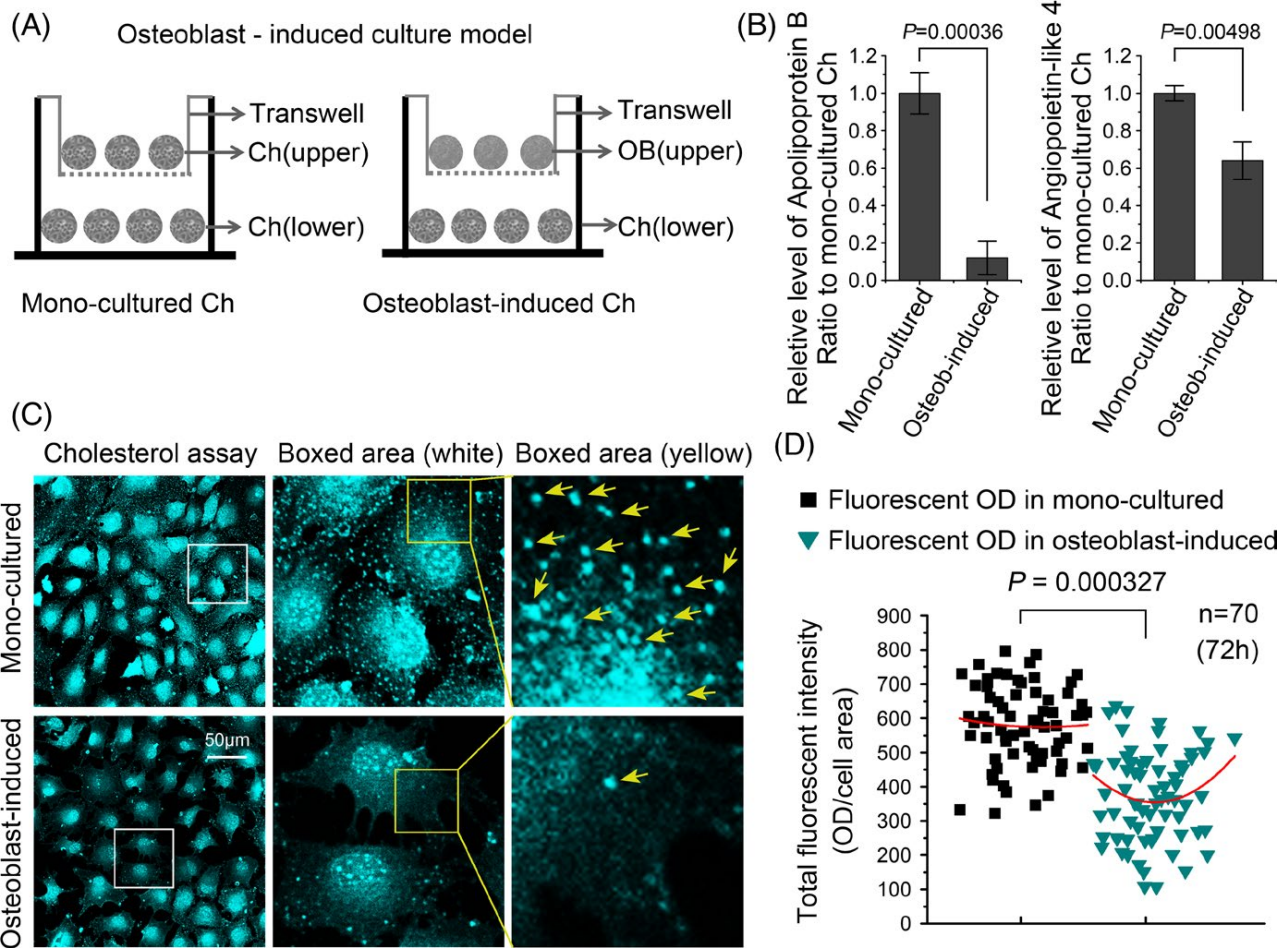
Osteoblast induction reduces the cholesterol level in chondrocytes. (A) Schematic diagram showing the osteoblast‐induced chondrocyte coculture model vs. chondrocyte monoculture model. (B) ELISAs showed that osteoblasts reduced apolipoprotein B and angiopoietin‐like 4 levels in chondrocytes (*n* = 3). (C) Filipin III staining shows the reduction in the cholesterol content in chondrocytes induced by osteoblasts compared with the monocultured chondrocytes (the yellow arrow indicates cholesterol particles in the chondrocyte cytoplasm). (D) Fluorescence OD measurement showing the change in the fluorescence intensity of cholesterol in chondrocytes in C. All results were obtained from three independent experiments (*n* = 3). The data in B are presented as the means ± SD. The analysis in D was based on 70 cells per group chosen from three independent experiments. Student's *t*‐test was applied to determine the significant differences in OD quantification in B and fluorescent OD quantification in D between the chondrocyte monoculture group and osteoblast‐induced chondrocyte coculture group

We established an osteoblast‐induced chondrocyte coculture model and compared it to a normal chondrocyte monoculture model to study the effects of osteoblasts on chondrocytes (Figure 1A). We first used ELISAs to detect the levels of apolipoprotein B (apoB), which represents the cholesterol content^18^ and angiopoietin‐like 4 (ANGPTL4), which inhibits lipid metabolism, to explore the effects of osteoblasts on chondrocyte lipid metabolism.^19^, ^20^ We observed a significant decrease in apoB and ANGPTL4 levels in chondrocytes induced by osteoblasts (apoB levels were decreased to 12.5% and ANGPTL4 levels were decreased to 65%) (Figure 1B). We obtained intuitive evidence for the effect of osteoblasts on chondrocyte cholesterol levels by measuring the fluorescence of Filipin III to show the changes in the cholesterol content in chondrocytes. We detected a significantly decreased cholesterol content in chondrocytes induced by osteoblasts (Figure 1C). The quantitative analysis of fluorescence further confirmed this decrease (Figure 1D).

### 3.2 Osteoblasts decrease the gene expression profile of the cholesterol metabolic pathway in chondrocytes

**FIGURE 2:**
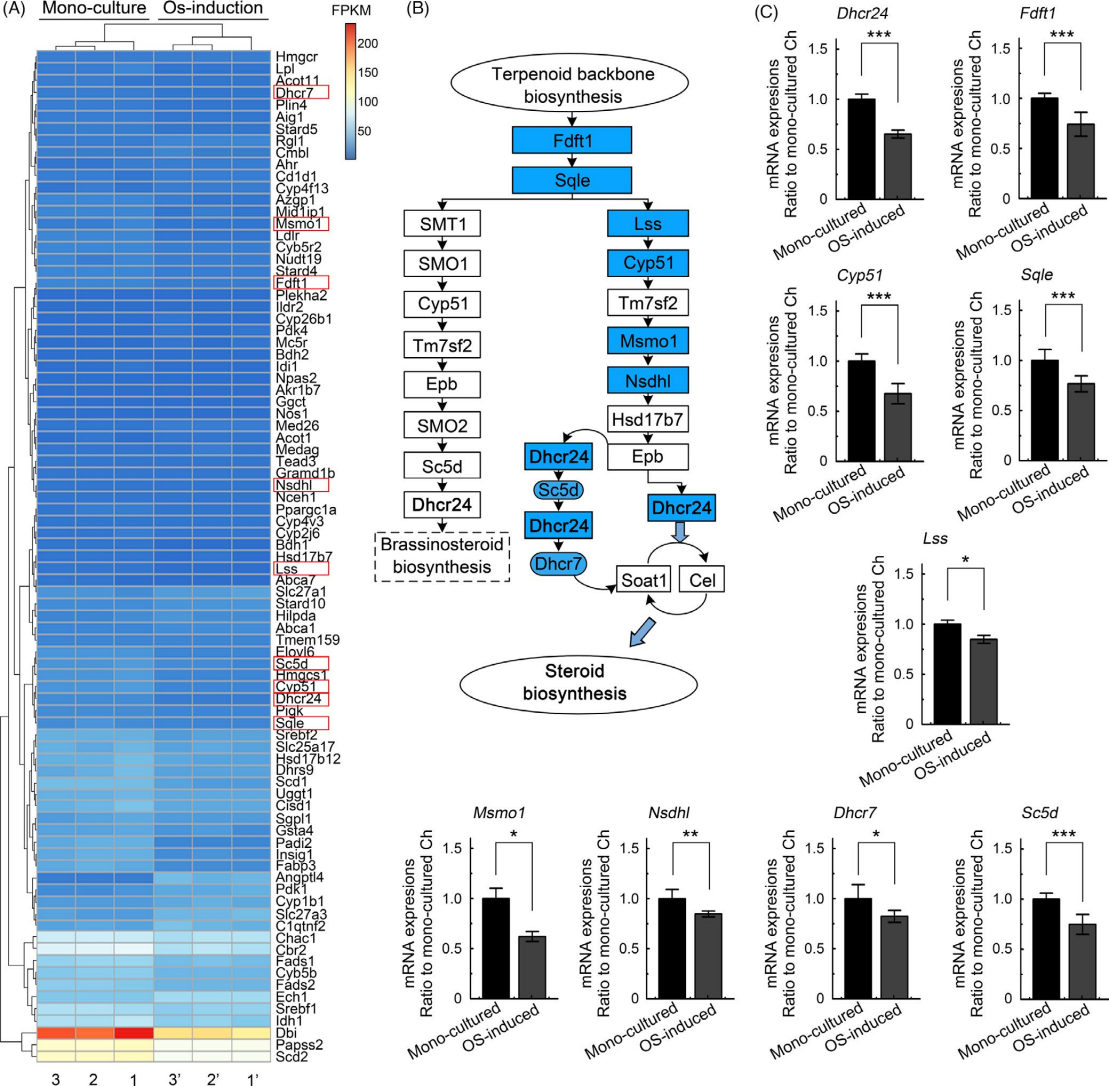
Osteoblasts induce the downregulation of gene expression profiles related to cholesterol synthesis in chondrocytes. (A) RNA sequencing showing the changes in the top 85 genes in the lipid metabolism‐related pathway (KEGG) between the osteoblast‐induced group and the chondrocyte monoculture group. The data are reported in FPKM and were formatted using the online R package. Three pairs of lysate samples were obtained from three independent cell isolates (*n* = 3). Each pair was derived from the same mother cells. Red boxes indicate the candidate genes participating in cholesterol synthesis. (B) Schematic diagram indicating the candidate genes in the steroid biosynthesis pathway (online KEGG database) identified in chondrocytes induced by osteoblasts. (C) qPCR showing changes in the expression of these candidate genes in chondrocytes induced by osteoblasts relative to monocultured chondrocytes. The expression is shown as the fold change relative to the internal GAPDH control. All results were obtained from three independent experiments (*n* = 3). The data in C are presented as the means ± SD, and Student's *t*‐test was applied to determine the significant differences between the chondrocyte monoculture group and the osteoblast‐induced chondrocyte coculture group. Cyp51, cytochrome P450, family 51; Dhcr24, 24‐dehydrocholesterol reductase; Dhcr7, 7‐dehydrocholesterol reductase; Fdft1, farnesyl diphosphate farnesyl transferase 1; FPKM, fragments per thousand bases of transcript for every one million fragments drawn; Lss, lanosterol synthase; Msmo1, methylsterol monooxygenase 1; Nsdhl, NAD(P)‐dependent steroid dehydrogenase‐like; Sc5d, sterol‐C5‐desaturase; Sqle, squalene epoxidase

We performed RNA sequencing to precisely determine the gene changes associated with lipid metabolism. We analysed the expression of all genes that showed changes and screened 86 lipid metabolism‐related genes in chondrocytes induced by osteoblasts, among which the expression of all genes related to the steroid synthesis pathway were substantially decreased (Figure 2A). By analysing the steroid synthesis pathway in the KEGG database, we identified 9 candidate genes in the steroid synthesis pathway of chondrocytes induced by osteoblasts (Figure 2B). We further confirmed the changes in the steroid synthesis pathway by performing qPCR. Changes in the expression of the farnesyl‐diphosphate farnesyltransferase 1 (Fdft1), squalene epoxidase (Sqle), lanosterol synthase (Lss), cytochrome P450, family 51 (Cyp51), methylsterol monooxygenase 1 (Msmo1), NAD(P)‐dependent steroid dehydrogenase‐like (Nsdhl), sterol‐C5‐desaturase (Sc5d), 7‐dehydrocholesterol reductase (Dhcr7) and 24‐dehydrocholesterol reductase (Dhcr24, also known as seladin‐1) mRNAs were all consistent with those identified using RNA sequencing (Figure 2C). We also showed the results for changes in the expression of genes in the brassinosteroid biosynthesis pathway and other steroid biosynthesis pathways (Figure S1), but the expression of these genes was not significantly changed.

### 3.3 Osteoblasts modulate the expression of the final synthetase, seladin‐1, in cholesterol synthesis in chondrocytes

**FIGURE 3:**
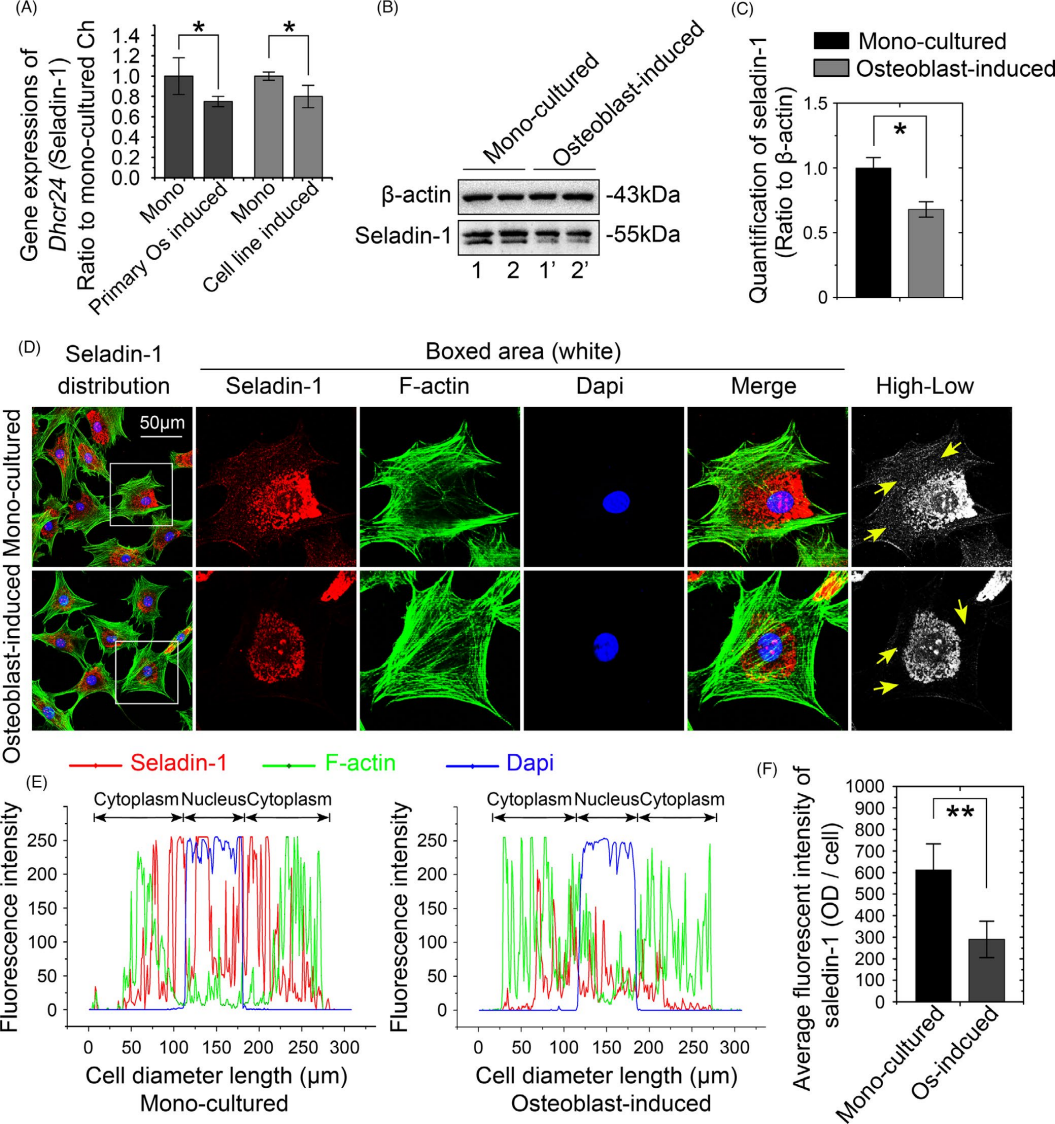
Osteoblasts induce a decrease in the cholesterol content in chondrocytes through the downregulation of seladin‐1. (A) qPCR showing the expression of the seladin‐1 mRNA in chondrocytes induced by osteoblasts relative to that in monocultured chondrocytes. We used both primary isolated osteoblasts and the osteoblastic MC3T3 cell line to treat chondrocytes and detect the expression of the seladin‐1 mRNA. (B) Representative western blots showing the reduced expression of seladin‐1 in chondrocytes induced by osteoblasts relative to that in monocultured chondrocytes. Samples 1 and 1′, 2 and 2′, were from the same mother chondrocytes. (C) Quantitative analysis confirming the changes in protein levels shown in B. (D) Immunofluorescence staining showing changes in the distribution of total seladin‐1 in chondrocytes induced by osteoblasts relative to the monocultured chondrocytes. Seladin‐1, red; cytoskeleton (FITC), green; nucleus (DAPI), blue. (E) Quantitative analysis of the linear distribution of seladin‐1 in chondrocytes induced by osteoblasts relative to that in monocultured chondrocytes. Seladin‐1, red; cytoskeleton (FITC), green; nucleus (DAPI), blue. (F) Histograms showing the changes in the total fluorescence intensity of seladin‐1 (per cell) in chondrocytes induced by osteoblasts relative to monocultured chondrocytes. All results were obtained from three independent experiments (*n* = 3). The data in A, C and F are presented as the means ± SD. Student's *t*‐test was applied to determine the significant differences between the chondrocyte monoculture group and the osteoblast‐induced chondrocyte coculture group. *p < 0.05; **p < 0.01

Seladin‐1 is a key factor involved in the last step of cholesterol synthesis.^21^ The results described above confirmed its expression at the mRNA level (Dhcr24, also known as seladin‐1, Figure 2A,C). Here, we used both primary osteoblasts and the MC3T3‐E1 cell line to induce seladin‐1 mRNA expression in chondrocytes growing in a Transwell chamber. The expression of the seladin‐1 mRNA was decreased in chondrocytes induced by primary osteoblasts and the MC3T3‐E1 cell line (Figure 3A). We then performed western blotting and found a significant decrease in the seladin‐1 protein level in chondrocytes after 72 h of induction by osteoblasts (Figure 3B,C). We assessed the changes in the seladin‐1 distribution in osteoblast‐induced chondrocytes by performing immunofluorescence staining. Seladin‐1 was present in the nucleus and cytoplasm (mostly around the nucleus, Figure 3D). After osteoblast‐induced treatment, seladin‐1 expression was decreased, especially in the cytoplasm (only a small amount of seladin‐1 was observed in the periphery of the cytoplasm, and detailed information is shown in the white boxed area) (Figure 3D). Linear fluorescence quantitative analysis showed changes in seladin‐1 levels in both the nucleus and cytoplasm (Figure 3E), and the final total fluorescence qualification confirmed that the changes in seladin‐1 expression in chondrocytes were induced by osteoblasts (Figure 3F).

### 3.4 Osteoblasts downregulate Notch1 signalling to mediate cholesterol synthesis in chondrocytes

**FIGURE 4:**
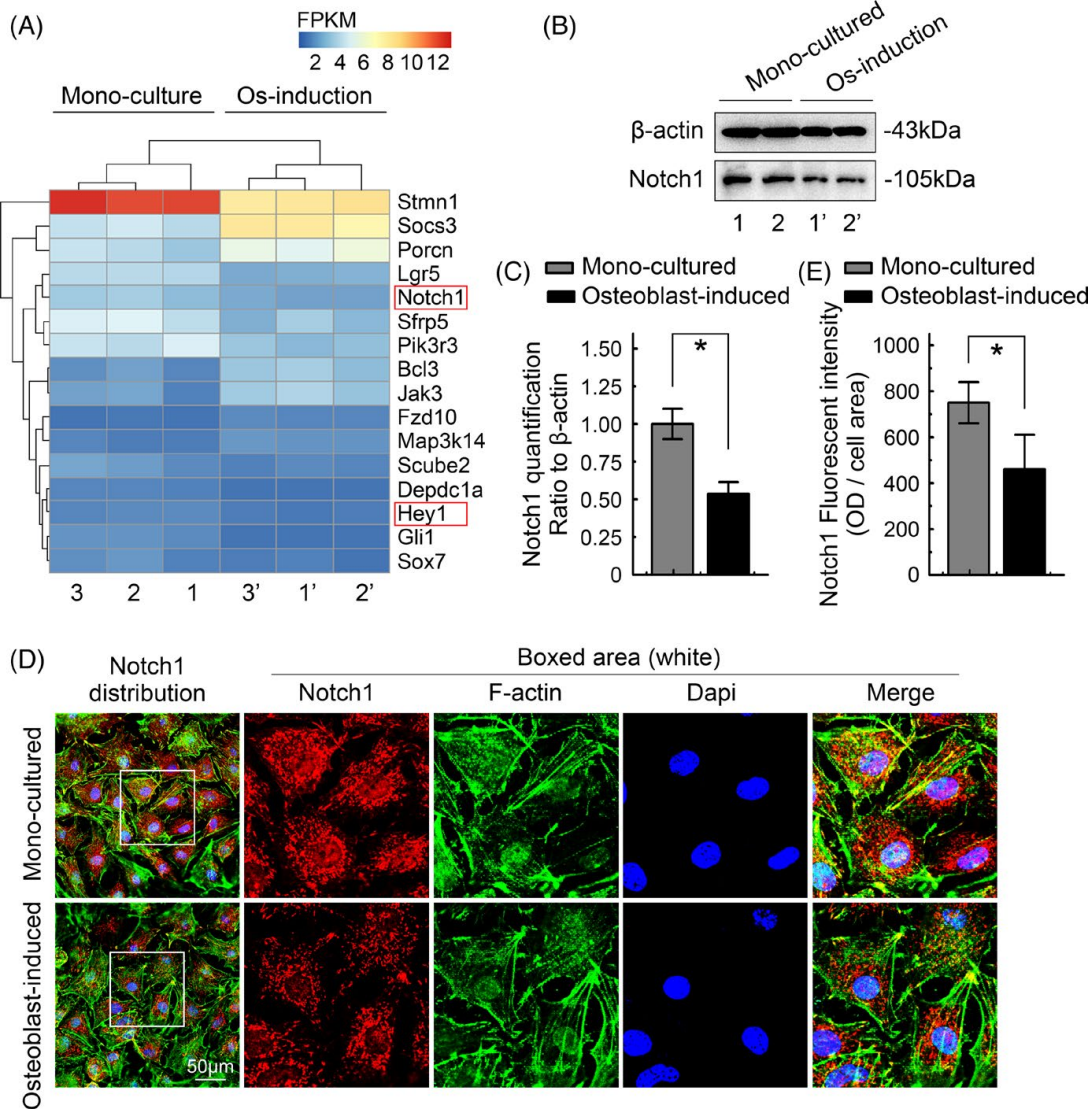
Osteoblasts decrease Notch1 signalling in chondrocytes. (A) RNA sequencing showing changes in the expression of the top 16 signalling pathway‐related genes and their downstream target genes (KEGG) in the osteoblast induction group and the chondrocyte monoculture group. The data are reported in FPKM and were formatted using the online R package. Three pairs of lysate samples were obtained from three independent cell isolates (*n* = 3), namely, samples 1 and 1′, samples 2 and 2′, and samples 3 and 3′. Each pair was derived from the same mother cells. Red boxes indicate Notch1 signalling and its downstream target gene hey1, which participate in osteoblasts to inhibit chondrocyte cholesterol metabolism. (B) Representative western blot showing decreased Notch1 expression in chondrocytes induced by osteoblasts. (C) Quantification of the changes in protein levels shown in B. (D) Representative CLSM images of IF staining showing the reduced expression and distribution of Notch1 in chondrocytes induced by osteoblasts. (E) Histograms showing the changes in the total fluorescence intensity of Notch1 (per cell) in chondrocytes induced by osteoblasts. All results were obtained from three independent experiments (*n* = 3). The data in B and D are presented as the means ± SD. Student's *t*‐test was applied to determine the significant differences between the chondrocyte monoculture group and the osteoblast‐induced chondrocyte coculture group. *p < 0.05. hey1, Hes‐related family bHLH transcription factor with YRPW motif 1; Notch1, Notch receptor 1

We analysed the RNA sequencing data and identified the downregulation of signalling pathways in chondrocytes induced by osteoblasts. From the pheatmap, we detected changes in the top 16 signalling pathway candidates in chondrocytes induced by osteoblasts (Figure 4A). Among them, Notch1 and its downstream target gene, Hes‐related family bHLH transcription factor with YRPW motif 1 (hey1), were significantly downregulated. The inhibition of canonical Notch signalling inhibits adipogenesis,^22^ whereas activation of this pathway stimulates adipogenesis.^23^ Thus, the Notch signalling pathway is considered an important regulator of chondrocyte cholesterol metabolism induced by osteoblasts. Using western blotting, we detected changes in the expression of Notch1 at the protein level, consistent with the results at the mRNA level (Figure 4B). Based on the quantitative analysis, the level of the Notch1 protein in chondrocytes was confirmed to be significantly decreased after osteoblast induction. Notch1 expression in osteoblast‐induced chondrocytes was reduced to as low as 50% relative to that in monocultured chondrocytes (Figure 4C). Using immunofluorescence staining and CLSM, we observed a decreased distribution of Notch1 in osteoblast‐induced chondrocytes (Figure 4D). Further quantification confirmed the total changes in Notch1 immunofluorescence in chondrocytes induced by osteoblasts (Figure 4E).

### 3.5 Osteoblasts decrease the expression of ncstn and hey1 to impair cholesterol synthesis in chondrocytes

**FIGURE 5:**
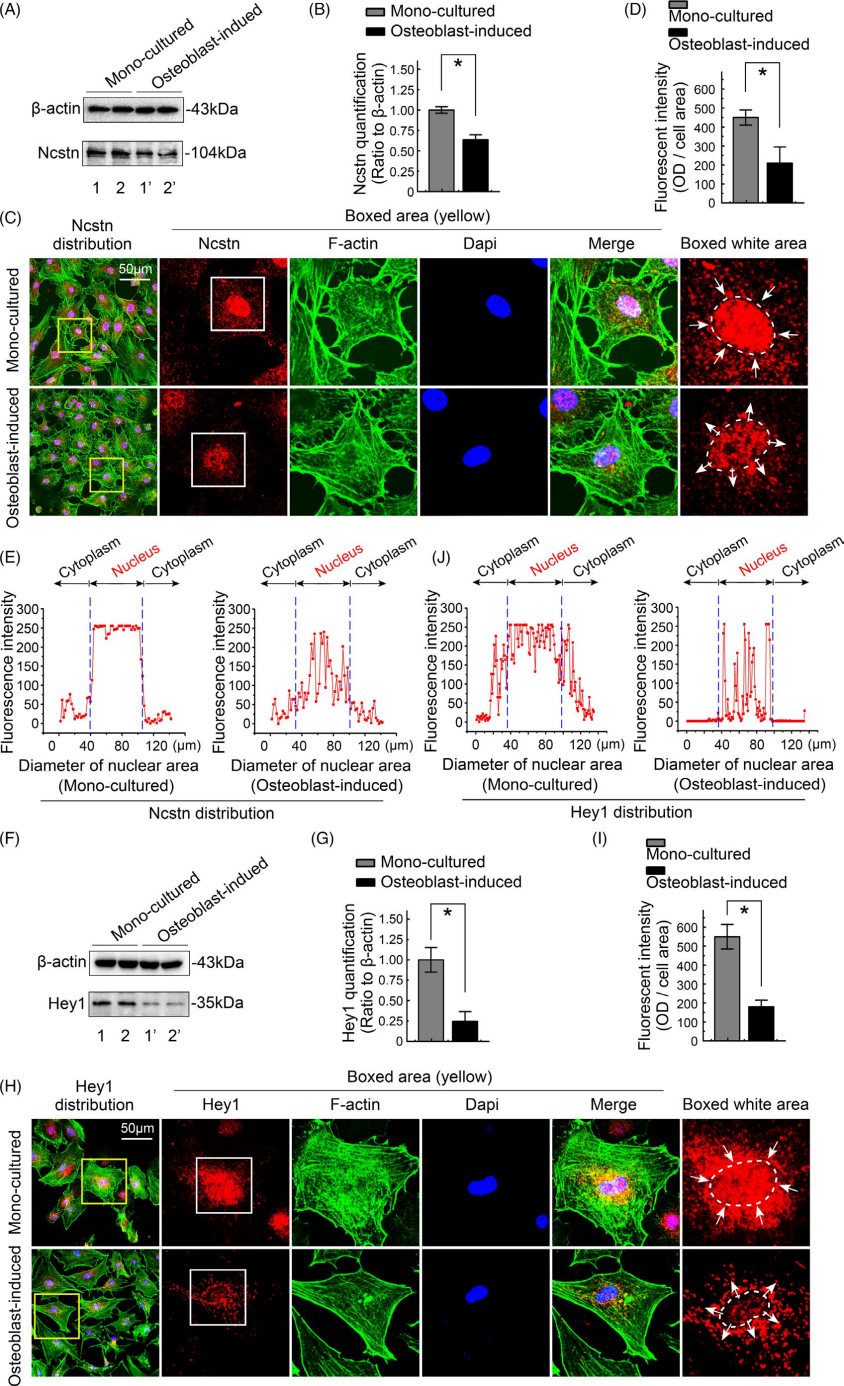
Osteoblasts induce a decrease in hey1 and ncstn expression in chondrocytes. (A) Western blot showing decreased ncstn expression in chondrocytes induced by osteoblasts. Samples 1 and 1′, 2 and 2′, were from the same mother chondrocytes. (B) Quantitative analysis of the changes in ncstn levels shown in A. (C) Representative CLSM images of IF staining showing the distribution of ncstn in chondrocytes induced by osteoblasts relative to monocultured chondrocytes. (D) Total fluorescence quantification was performed to show the changes in ncstn levels in chondrocytes. (E) Linear fluorescent quantification was performed to show the intracellular distribution of ncstn in chondrocytes. (F) Western blot showing decreased expression of hey1 in chondrocytes induced by osteoblasts. Samples 1 and 1′, 2 and 2′, were from the same mother chondrocytes. (G) Quantitative analysis of the changes in hey1 levels shown in F. (H) Representative CLSM images of IF staining showing the distribution of hey1 in chondrocytes induced by osteoblasts relative to that in monocultured chondrocytes. (I) Total fluorescence quantification was performed to show the changes in hey1 expression in chondrocytes. (J) Linear fluorescent quantification was performed to show the intracellular distribution of hey1 in chondrocytes. All results were obtained from three independent experiments (*n* = 3). The data in B, D, G and I are presented as the means ± SD. Student's *t*‐test was applied to determine significant differences between the chondrocyte monoculture group and the osteoblast‐induced chondrocyte coculture group. *p < 0.05. hey1, Hes‐related family bHLH transcription factor with YRPW motif 1; Ncstn, nicastrin

We aimed to further determine the importance of Notch1 signalling in chondrocytes induced by osteoblasts by specifically assessing the expression of ncstn, an activator of Notch signalling, and hey1, a specific downstream target of Notch signalling. Western blots showed changes in ncstn levels in chondrocytes induced by osteoblasts (Figure 5A). Quantitative analysis confirmed the significant decrease in ncstn levels in chondrocytes induced by osteoblasts (Figure 5B). We then showed the change in its distribution in chondrocytes induced by osteoblasts by performing immunofluorescence staining. The expression of ncstn was decreased in both the cytoplasm and nucleus of chondrocytes (Figure 5C). Total fluorescence quantification showed a decreased amount of ncstn in chondrocytes induced by osteoblasts (Figure 5D). According to the linear fluorescence quantification, we observed a dramatic reduction in ncstn levels in the nuclear region of chondrocytes induced by osteoblasts (Figure 5E). At the same time, we detected hey1 levels using western blotting and observed a significant decrease in its levels in chondrocytes induced by osteoblasts (Figure 5F,G). Using CLSM, we detected a substantial decrease in hey1 levels in the nuclear region (Figure 5H). Total fluorescence quantification showed a decreased amount of hey1 in chondrocytes induced by osteoblasts (Figure 5I). Based on the linear fluorescence quantification, we observed a dramatic reduction in hey1 levels in the nuclear region of chondrocytes induced by osteoblasts (Figure 5J). Taken together, these results indicate the potential role of Notch1 signalling in mediating osteoblast‐induced cholesterol synthesis in chondrocytes.

## 4 DISCUSSION

Previous research has shown crosstalk between osteoblasts and chondrocytes. In the process of endochondral ossification, chondrocytes proliferate and differentiate into hypertrophic chondrocytes, which are invaded by blood vessels and replaced by osteoblasts after apoptosis. In this process, chondrocytes and osteoblasts interact precisely through direct contact or paracrine pathways.^3^, ^24^ Cholesterol is an indispensable structural component of cell membranes and is essential for the biosynthesis, integrity and function of biofilms. In addition, cholesterol is also a precursor of various hormones, such as adrenal cortex hormones, oestrogen and androgen and progesterone.^25^ Cholesterol also plays an important role in the process of cartilage formation,^26^ and abnormal cholesterol accumulation in chondrocytes is related to the severity of OA.^11^ However, the effect of osteoblasts on chondrocyte cholesterol metabolism and its underlying mechanism remains unclear.

**FIGURE 6:**
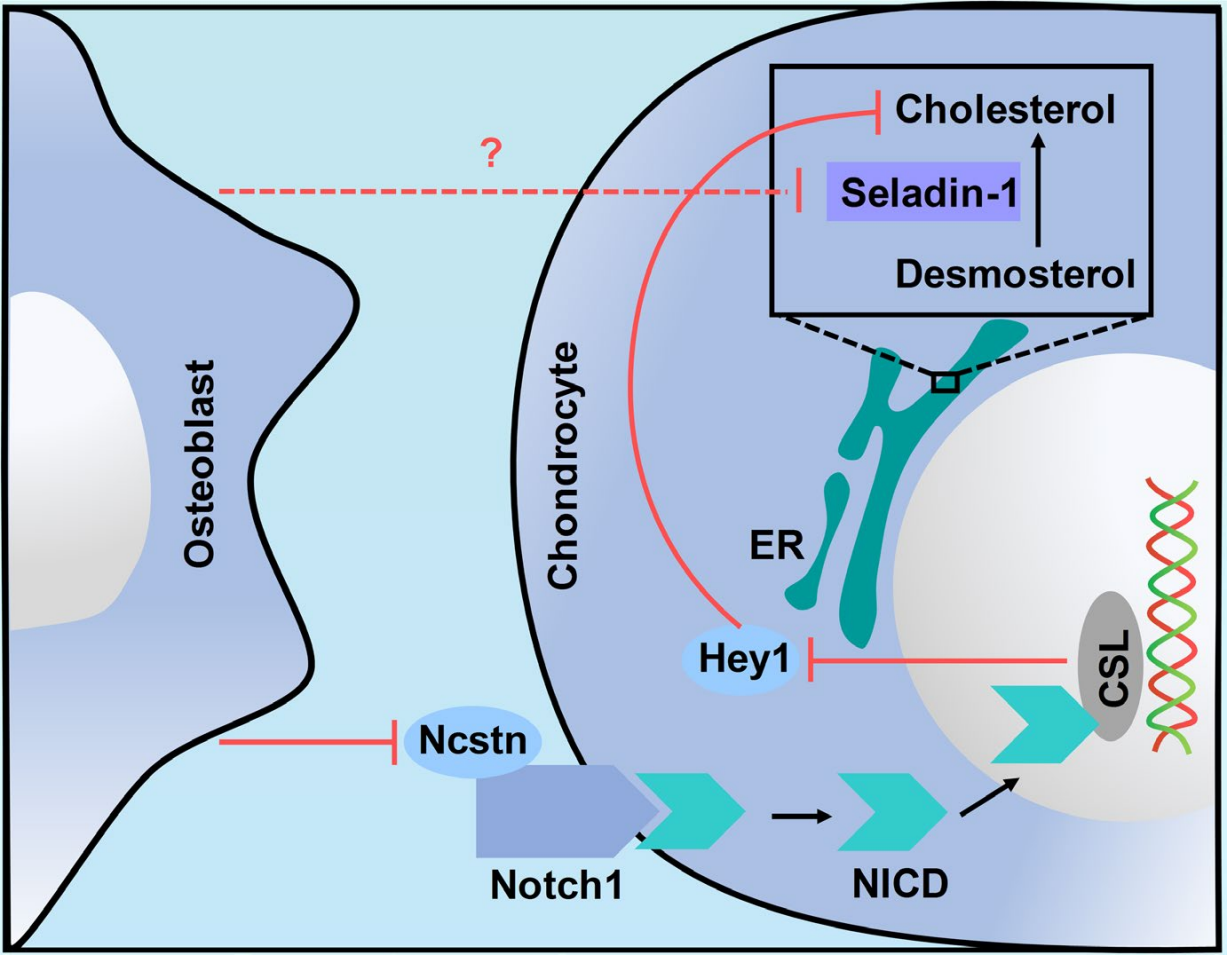
Schematic diagram illustrating the mechanism of osteoblast‐induced regulation of cholesterol synthesis in chondrocytes. The red lines point to the Notch1 signalling pathway elucidated in the current study, and the red dotted lines indicate potential mechanisms that were not assessed in the present study. Osteoblasts inhibited ncstn expression in chondrocytes and reduced its binding to Notch receptors, resulting in a decrease in the expression of hey1 downstream of the Notch signalling pathway. Finally, cholesterol metabolism in chondrocytes was inhibited. ER, endoplasmic reticulum; hey1, Hes‐related family bHLH transcription factor with YRPW motif 1; ncstn, nicastrin

Osteoblasts inhibit the release of the Notch protein fragment NICD with transcriptional regulatory activity, and its binding to the transcription factor CSL is blocked, which reduces the expression of the hey1 gene downstream of the pathway and inhibits the cholesterol synthase seladin‐1, thereby inhibiting the synthesis of cholesterol in chondrocytes. (Figure 6). ELISAs confirmed that osteoblasts reduced the expression of apoB and ANGPTL4 in chondrocytes. ApoB, which is usually present in the form of apolipoprotein B100 and apolipoprotein B48, is a large amphiphilic protein.^27^ It is located on the surface of low‐density lipoprotein. ApoB mainly recognizes and promotes the uptake of LDL by cells, and thus it is an indispensable part of lipoprotein metabolism. Since LDL is a lipoprotein enriched in cholesterol, the apoB content potentially reflects the cholesterol content.^18^ ANGPTL4 is also called peroxisome proliferator‐activated receptor‐γ, hepatic fibrinogen or fasting‐induced adipokine.^28^ The main role of ANGPTL4 is to regulate lipid metabolism and angiogenesis. The role of ANGPTL4 in lipid metabolism is to inhibit lipoprotein lipase activity, increase plasma triacylglycerol and nonesterified fatty acid levels and then reduce adipose tissue reserves.^21^ Another study found that ANGPTL4 is only expressed at low levels in normal cartilage, and the amount of ANGPTL4 also represents the number of cartilage cells to a certain extent.^28^ In summary, we propose that the level of this protein may indicate a decrease in cholesterol levels. Therefore, we used Filipin III to specifically stain cholesterol and found that the cholesterol content in chondrocytes was indeed reduced after osteoblast induction.

Steroid compounds are widely found in animals and plants, and they play extremely important roles in regulating biological activities in the lives of animals and plants.^29^, ^30^, ^31^, ^32^ Among these steroids, cholesterol is the most important sterol in animals. We sequenced the top 86 genes in the lipid metabolism‐related pathway (KEGG) showing altered expression between the osteoblast induction group and the chondrocyte monoculture group to explore the mechanism by which osteoblasts reduced cholesterol levels in chondrocytes and identified 9 genes involved in the steroid biosynthetic pathway showing consistent changes. This phenomenon may indicate that the induction of osteoblasts inhibits the entire cholesterol synthesis pathway in chondrocytes. Based on the qPCR results, seladin‐1 showed the most significant change. Seladin‐1 catalyses the conversion of desmosterol to cholesterol, which is the ultimate step in cholesterol biosynthesis.^33^, ^34^ Seladin‐1 also plays an important role in bones and joints. For instance, desmosterolosis, an autosomal recessive genetic disease caused by mutations in the seladin‐1 gene, is associated with abnormal bone development. Mirza et al.^35^ utilized seladin‐1 knockout mice and found that seladin‐1 plays an important role in long bone growth by protecting chondrocytes from ROS. The results of our western blot analysis also confirmed that seladin‐1 levels in chondrocytes were reduced by osteoblasts. Accordingly, we speculate that osteoblasts inhibit the progress of cholesterol synthesis by inhibiting seladin‐1. According to the CLSM results, seladin‐1 was primarily located in the nucleus following osteoblast induction, while the level of seladin‐1 in the cytoplasm was significantly reduced (Figure 3D,E). This change is similar to the phenomenon mentioned in a previous study: seladin‐1, which is normally confined to the perinuclear cytoplasmic region, is localized in the nuclei after oxidative challenge.^36^ Consequently, we speculate that induction by osteoblasts may cause oxidative stress in chondrocytes. In other words, the reduction in chondrocyte lipid synthesis may correlate with its oxidative stress state. Further research is needed to prove this hypothesis.

Notch1 signalling is closely related to lipid metabolism. Our RNA sequencing data also confirmed that Notch1 exerts its biological effects on the process by which osteoblasts inhibit cholesterol synthesis in chondrocytes. Ncstn, also referred to as nicastrin, is located on chromosome 1q22–q23 and is required for γ‐secretase cleavage of the Notch receptor to activate the Notch pathway.^37^, ^38^ Hey1 is an important downstream target of the Notch signalling pathway, and recent studies have shown that a reduction in hey1 expression obstructs adipogenesis.^39^ In the present study, we performed Western blot analyses and found that the expression of ncstn and hey1 in chondrocytes decreased after osteoblast induction. Accordingly, we speculate that osteoblasts inhibit Notch1 signalling in chondrocytes by inhibiting ncstn and hey1. Additionally, osteoblast‐induced culture also affected the subcellular localization of hey1; CLSM revealed that hey1 migrates towards the margin of the nucleus. Hey1 was mainly located in the nucleus of normal chondrocytes, where it regulated transcriptional activity, indicating the activation of the Notch signalling pathway. After osteoblast induction, hey1 began to escape from the nucleus and its transcriptional activity decreased, and Notch1 signalling was inhibited. Interestingly, this result is similar to the characteristics of hey1 mentioned in a previous study. In the control group, hey1 showed uniform nuclear localization. After 6 h of treatment with actinomycin D, hey1 migrated to the edge of the nucleolus.^40^ Actinomycin D is a ribosomal stress‐inducing agent, and ribosomal stress is related to the cell cycle, apoptosis and other processes.^41^ We speculate that osteoblasts may exert this effect on chondrocytes. More detailed studies are needed to confirm this assumption.

In summary, our research is the first to reveal the molecular mechanism by which osteoblasts inhibit cholesterol synthesis in chondrocytes and confirms that osteoblasts play a regulatory role through Notch1 signalling and seladin‐1. This research has improved our comprehension of the molecular mechanisms by which osteoblast‐derived signals regulate chondrocyte behaviour.

## AUTHOR CONTRIBUTIONS

Jing Xie and Xuedong Zhou designed the experiments. Yueyi Yang, Daimo Guo, Jiachi Li, Siqun Xu and Jiaya Wei performed the all experiments. Yueyi Yang, Demao Zhang and Jing Xie analysed and confirmed all data and prepared the manuscript. All authors reviewed the manuscript, and Jing Xie and Xuedong Zhou made a final approval.

## CONFLICT OF INTEREST

The authors have no competing interests to declare.

## Supporting information

Figure S1

Data S1

## Data Availability

Any data generated in this study are available from the corresponding author upon request.
