# Supplementary material for: Osteoblasts impair cholesterol synthesis in chondrocytes via Notch1 signalling
Source: Cell Prolif. 2021 Nov 2;54(12):e13156. doi: 10.1111/cpr.13156 (PMC8666287; doi:10.1111/cpr.13156)
Supplement: Supplementary file 1 — Figure S1 [file CPR-54-e13156-s001.doc]

Supplementary figures for article:

**Osteoblast inhibit cholesterol metabolism of chondrocyte via Notch 1 signaling**

**Supplementary figures and legnends**

**Figure S1**


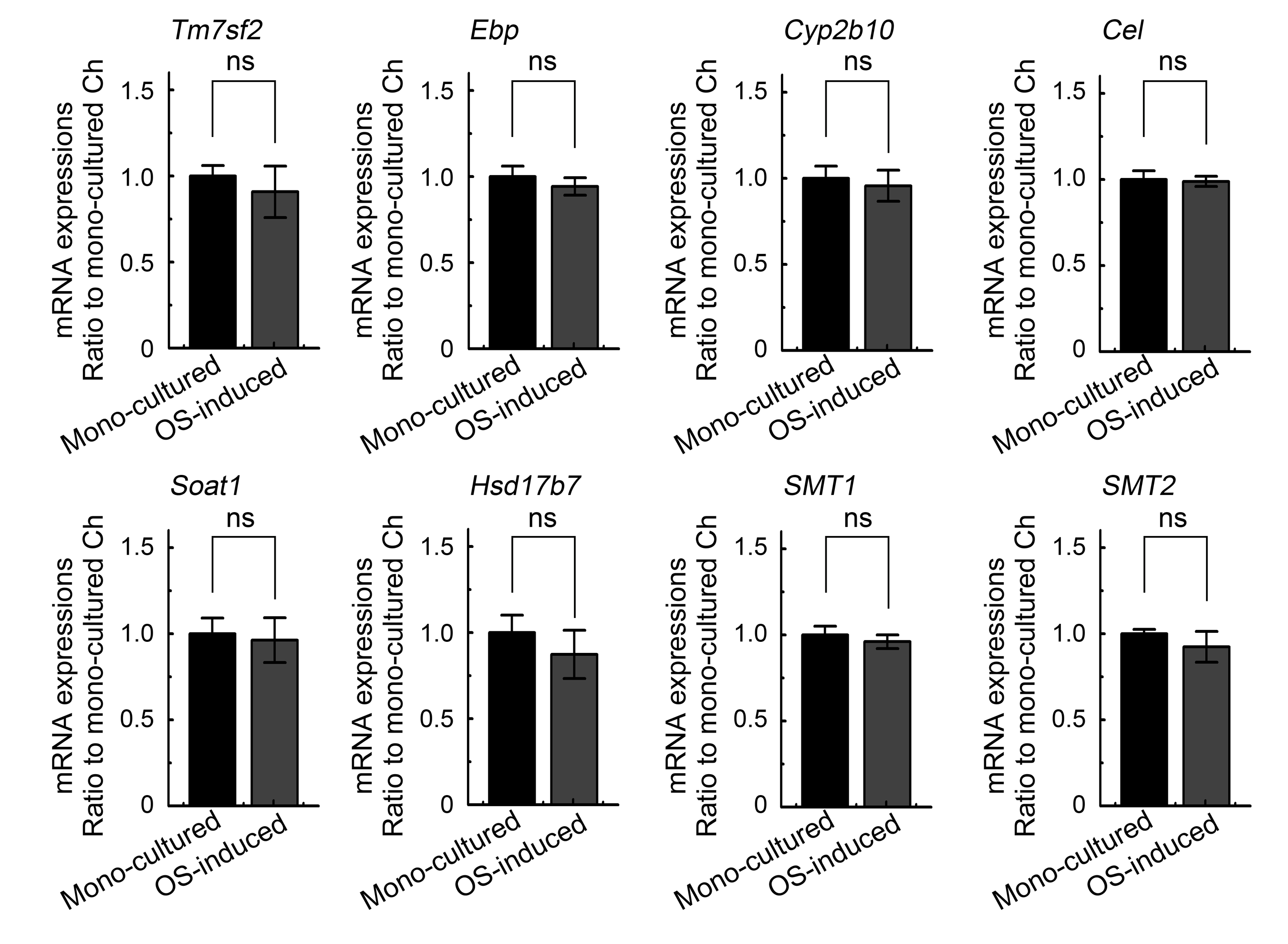


**Figure S1. The unchanged genes of brassinosteroid biosynthetic pathway and steroid biosynthetic pathway in chondrocytes induced by osteoblasts.**

qPCR showed the expression of *Tm7sf2*, *Ebp*, *Cyp2b10*, *Cel*, *Soat1*, *Hsd17b7*, *SMT1* and *SMT2* in chondrocytes induced by osteoblasts. The gene profiles of these members were calculated by normalizing to internal β-actin. Data were derived from the three independent experiments (n = 3). Student T tests were performed to examine the differences between mono-cultured chondrocytes and osteoblast-induced chondrocytes.
